# In Vitro Phase I Metabolism of CRV431, a Novel Oral Drug Candidate for Chronic Hepatitis B

**DOI:** 10.3390/pharmaceutics9040051

**Published:** 2017-11-09

**Authors:** Daniel J. Trepanier, Daren R. Ure, Robert T. Foster

**Affiliations:** ContraVir Pharmaceuticals Inc., Edison, NJ 08837, USA; dtrepanier@contravir.com (D.J.T.); dure@contravir.com (D.R.U.)

**Keywords:** cyclophilin inhibitor, CRV431, P450, metabolism, species differences

## Abstract

The cytochrome P450-mediated Phase I in vitro metabolism of CRV431 was studied using selective chemical inhibition and recombinant human enzymes. Additionally, the metabolic profile of CRV431 in human, rat, and monkey liver microsomes was investigated. Liver microsomes were incubated for 0–80 min with CRV431, and the metabolite profile was assessed by electrospray ionization liquid chromatography mass spectrometry (ESI-LCMS). CRV431 was extensively metabolized through oxidation to produce various hydroxylated and demethylated species. Species identified included monohydroxylated CRV431 (two distinct products), dihydroxylated CRV431, demethylated CRV431 (two distinct products), demethylated and hydroxylated CRV431 (two distinct products), didemethylated and hydroxylated CRV431, and didemethylated and dihydroxylated CRV431. The magnitude of metabolism was greatest in monkey, followed by human, followed by rat. Importantly, all of the species identified in human microsomes were correspondingly identified in monkey and/or rat microsomes. Human liver microsome studies using selective chemical inhibition, as well as studies using recombinant human cytochrome P450 enzymes, revealed that the major enzymes involved are cytochromes P450 3A4 and 3A5. Enzymes 1A2, 2B6, 2C8, 2C9, 2C19, and 2D6 are not involved in the in vitro metabolism of CRV431. This information will be useful for the further development of CRV431 both preclinically and clinically.

## 1. Introduction

Cyclosporine A (CsA) was isolated from *Tolypocladium inflatum* in 1971, and has been used clinically since the early 1980s as an immunosuppressive drug to prevent rejection after solid organ transplantation [1]. CsA and other immunosuppressive analogues bind to cyclophilin, and the complex further binds and inhibits the activity of calcineurin, a phosphatase that activates T cells [2].

A novel investigational drug that is a non-immunosuppressive derivative of CsA, CRV431, is currently under development. The immunosuppressive property of CsA has been removed by modification of the CsA undecapeptide scaffold by introducing chemical modifications on amino acids 1 and 3. This alteration to the CsA backbone has been made to exploit the known properties of cyclophilins in normal physiology and in disease. Cyclophilins play an important role in infectious diseases, including for example, treatment of the hepatitis C virus (HCV) and hepatitis B virus (HBV). CRV431 has been shown previously to block replication of HBV, HCV, and human immunodeficiency virus type 1 (HIV-1) in vitro by inhibiting important interactions of the viruses with host cell cyclophilins [3,4,5]. CRV431 is currently being studied as a host-targeting investigational drug to treat chronic HBV. In in vitro [4,5] and in vivo (*unpublished data*) studies to date, CRV431 has been shown to reduce HBV and DNA, as well as target HBV proteins, including protein X (HBx) and surface antigen (HBsAg), through its ability to abrogate binding of cyclophilins to proteins. It is anticipated that CRV431 will complement the landscape of direct acting anti-HBV drugs such as tenofovir and entecavir.

CsA undergoes extensive hepatic metabolism with elimination primarily through biliary excretion. Less than 10% of the intact drug and metabolite are excreted in urine. Cytochrome P-450 3A has been identified as the major metabolizing enzyme [6,7,8], which results in multiple primary hydroxylations and *N*-demethylations, along with secondary and tertiary products.

Here, we describe the cytochrome P450-mediated Phase I in vitro metabolism of CRV431 using selective chemical inhibition and recombinant human enzymes to gain a better understanding of the fate of the parent molecule, and understand potential routes of elimination of both parent and metabolite. Additionally, we investigate the metabolic profile of CRV431 in human, rat, and monkey liver microsomes, and identify multiple metabolites by liquid chromatography—mass spectrometry (LC-MS).

## 2. Materials and Methods

### 2.1. Drugs and Reagents

CRV431 was synthesized in-house to a purity of 97.3% by modification of cyclosporin A and stored at 5 °C. CsA was obtained from *IVAX* (Czech Republic). Human and rat liver microsomes, as well as recombinant human CYP enzymes, were obtained from Sekisui Xenotech, Kansas City, MO, USA. Monkey liver microsomes were obtained from Thermo Fisher Scientific, Waltham, MA, USA. All chemicals and reagents were purchased from Sigma-Aldrich, St. Louis, MO, USA.

### 2.2. Metabolic Stability of CRV431 in Human Liver Microsomes

Incubations of CRV431 (1 and 10 μM) with pooled human liver microsomes (1 mg protein/mL) were conducted for 30 min. Incubations were performed at 37 ± 1 °C in 0.2 mL incubation mixtures (final volume) containing potassium phosphate buffer (50 mM, pH 7.4), MgCl_2_ (3 mM) and EDTA (1 mM, pH 7.4) with an NADPH-generating system as cofactor. The NADPH-generating system consisted of NADP (1 mM, pH 7.4), glucose-6-phosphate (5 mM, pH 7.4), and glucose-6-phosphate dehydrogenase (1 Unit/mL). CRV431 was added to incubations in DMSO (0.1% *v*/*v*). Additional incubations of CRV431 (1 and 10 μM) with human liver microsomes (1 mg protein/mL) were carried out at 0, 15, 30, 60, and 120 min. Reactions were initiated by the addition of the NADPH-generating system, and were stopped by the addition of 175 μL of acetonitrile. The samples were centrifuged (920× *g* for 10 min at 10 °C), and the supernatant fractions were analyzed by LC-MS/MS (Sciex, Redwood City, CA, USA) to quantify the amount of unchanged CRV431 based on a calibration curve (ranging from 0.01 to 15 μM). Zero-time, zero-cofactor (no NADPH), zero-substrate, and zero-protein served as blanks.

### 2.3. Cytochrome P450 Metabolism of CRV431 Using Recombinant Human CYP Enzymes

This experiment was carried out to determine the cytochrome P450 (CYP) enzymes capable of metabolizing CRV431. Briefly, CRV431 at two concentrations (1 and 10 μM) was incubated in duplicate with a panel of recombinant human CYP enzymes (rCYP1A2, 2B6, 2C8, 2C9, 2C19, 2D6, and 3A4 at 50 pmol CYP/mL) at 37 ± 1 °C in 0.2 mL incubation mixtures (final volume) containing potassium phosphate buffer (50 mM, pH 7.4), MgCl_2_ (3 mM) and EDTA (1 mM, pH 7.4) with an NADPH-generating system as cofactor. The NADPH-generating system consisted of NADP (1 mM, pH 7.4), glucose-6-phosphate (5 mM, pH 7.4), and glucose-6-phosphate dehydrogenase (1 Unit/mL). CRV431 was added to the incubation mixtures in DMSO (0.1% *v*/*v*). Reactions were initiated by the addition of cofactor, and were terminated at zero and 15 min by the addition of 175 μL ice-cold methanol. After the reactions were stopped, samples were analyzed by LC-MS/MS to quantify the amount of unchanged CRV431.

### 2.4. Cytochrome P450 Metabolism Using Chemical Inhibition in Human Liver Microsomes

This experiment was carried out manually to verify the role of individual CYP enzymes in the metabolism of CRV431. CRV431 at 1 μM was incubated in duplicate with human liver microsomes (1 mg protein/mL) for zero and 15 min in the presence and absence of the chemical inhibitors summarized in the table below. Incubations were conducted at 37 ± 1 °C in 0.2 mL incubation mixtures (final volume) containing potassium phosphate buffer (50 mM, pH 7.4), MgCl_2_ (3 mM) and EDTA (1 mM, pH 7.4), with an NADPH-generating system as cofactor. The NADPH-generating system consisted of NADP (1 mM, pH 7.4), glucose-6-phosphate (5 mM, pH 7.4), and glucose-6-phosphate dehydrogenase (1 Unit/mL). CRV431 was added to the incubation mixtures in DMSO (0.1% *v*/*v*). For incubations with direct-acting chemical inhibitors, reactions were initiated by the addition of cofactor, and stopped by the addition of an equal volume of acetonitrile stop reagent. For incubations with metabolism-dependent chemical inhibitors, 30 min pre-incubations of human liver microsomes and the inhibitor were initiated by the addition of the cofactor. CRV431 was then added to the mixture to be incubated for 15 min. Reactions were stopped by the addition of an equal volume of acetonitrile stop reagent. Samples were analyzed by LC-MS/MS to quantify the amount of unchanged CRV431. Control samples with no CYP inhibitor present were conducted in the presence of the solvent used to dissolve the CYP inhibitor (“solvent control”).

### 2.5. LC-MS/MS Analysis of CRV431 for Cytochrome P450 Studies

Samples were analyzed by LC-MS/MS on a SCIEX 4000 QTrap mass spectrometer (Sciex, Rewood City, CA, USA). CRV431 samples were injected onto an Acquity UPLC BEH C18 analytical column (50 × 2.1 mm, 1.7 μm, Waters, Milford, MA, USA) and separated using a methanol-water gradient system (Table 1). The ammonium adducts of CRV431 (mass transition 1321.5/1304.5) were analyzed by mass spectrometry using electrospray ionization (ESI) in positive ion mode. The electrospray voltage was set at 4500 volts.

### 2.6. Microsome Incubation Procedure for Generation of CRV431 Metabolites

Human and rat liver microsomes were obtained from Xenotech (Kansas City, MO, USA) and stored at −80 °C. Monkey liver microsomes were obtained from Thermo Fisher Scientific (Waltham, MA, USA) and stored at −80 °C. Preliminary microsomal biotransformation experiments were run with 0.1, 1, and 10 μg/mL CRV431. CRV431 biotransformation was minimal at 0.1 μg/mL, while both 1 and 10 μg/mL CRV431 gave sufficient metabolite quantity and identical metabolite profiles. Additionally, preliminary experiments were conducted with human liver microsome (HLM) levels ranging for 0.125 to 1 mg. Subsequent microsomal biotransformation experiments were conducted using 0.5 mg of microsome per assay.

Thawed microsome 20 mg/mL stocks were diluted 32× into cold buffer (Phosphate pH 7.4 + MgCl2 3 mM + EDTA 1 mM) to 0.625 mg/mL, and stored on ice. For experiments with CRV431, 2 μL of 0.625 mg/mL CRV431 stocks were added to cold 1-mL microsomes (500× dilution) to generate the 1 μg/mL samples. For experiments with verapamil, 2 μL of 625 μM was added to cold 1-mL human microsomes (500× dilution). Samples were mixed by vortexing, and 160 μL was transferred into tubes containing 40 μL buffer on ice. Metabolic activity was stopped by the addition of ice-cold methanol (200 μL), followed by vortexing for an additional 10 s; these were zero-time samples. The extraction was completed by waiting 5 min, vortexing samples again for 5 s, then spinning the samples for 10 min at 3300 rpm at 4 °C in a pre-cooled microcentrifuge. The supernatant (325 μL) was transferred to new tubes and stored at −80 °C.

For time course experiments, metabolism was initiated by adding 40 μL of NADPH Regenerating System (5 mg/mL NADP, 6.5 mg/mL G6P and 5 units/mL of G6P dehydrogenase). The sample was vortexed for 2 s and placed in a 37 °C bath. For CRV431 experiments at time points 10 min, 20 min, 40 min, and 80 min, 200 μL of ice-cold methanol was added to the 200 μL microsome reactions, and extracted as described above. Samples were stored at −80 °C. For verapamil experiments at 15 min, 200 μL of ice-cold methanol was added to the 200 μL microsome reactions and extracted as described above. Samples were stored at −80 °C.

### 2.7. CRV431 and Metabolite Extraction from Rat, Monkey and Human Microsomes

Microsome extracts were removed from the freezer (−80 °C) and left to thaw at room temperature. Aliquots (50 μL) were removed and placed in 2 mL plastic Eppendorf microcentrifuge tubes, to which 200 μL of a 0.2 M ZnSO_4_ solution (0.2 M ZnSO_4_ in water, plus HPLC grade methanol, 1:4, *v*/*v*) was added as protein precipitating reagent. The tubes were capped and vortexed for 10 s, and then left on the benchtop for 10 min. Samples were then centrifuged at 3300 rpm for 10 min. The clear supernatant (≈150 μL) was transferred to an appropriately labeled LC-MS autosampler vial and capped.

### 2.8. LC-MS Analysis of CRV431 Metabolites

Samples were analyzed by electrospray ionization liquid chromatography mass spectrometry (ESI-LCMS) on an Agilent HP 1100 LC-MS. Samples were placed in an autosampler maintained at 5 °C. CRV431 microsomal extracts (20 μL) were injected onto a Zorbax SB-C18 reverse phase HPLC column (1.8 μm Rapid Resolution HT Cartridge, 4.6 × 30 mm (Agilent, Santa Clara, CA, USA) maintained at 75 °C, and the components were separated using an acetonitrile–water gradient system (Table 2). The sodium adducts of CRV431 (1326 *m*/*z*) and metabolites were analyzed by mass spectrometry (MS) using electrospray ionization (ESI) in positive ion mode. The ESI–MS was optimized with N_2_ gas temperature set at 350 °C and drying gas at 12/L min. The fragmentor and capillary voltages were set at 260 and 4000 volts, respectively. The nebulizer pressure was set at 40 psig. For the initial analysis of the microsomal extracts, the ESI–MS was run in scan mode (1260–1460 *m*/*z*) to capture all ion signals present. Subsequently, the microsomal extracts were run in selected ion mode (SIM), with the ions identified in the scans.

### 2.9. Verapamil LC-MS Analysis for Microsome Viability

Samples were analyzed by electrospray ionization liquid chromatography mass spectrometry (ESI-LCMS) on an Agilent HP 1100 LC-MS (Agilent, Santa Clara, CA, USA). Samples were placed in an autosampler maintained at 5 °C. Verapamil microsomal extracts (2 μL) were injected onto a Zorbax SB-C18 reverse phase HPLC column (1.8 μm Rapid Resolution HT Cartridge, 4.6 × 30 mm) maintained at 75 °C, and the verapamil was separated using an acetonitrile–water gradient system (Table 3). The sodium adduct of verapamil (455.5 *m*/*z*) was analyzed by mass spectrometry (MS) using electrospray ionization (ESI) in positive ion mode. The ESI–MS was optimized with N_2_ gas temperature set at 350 °C and drying gas at 12/L min. The fragmentor and capillary voltages were set at 150 and 4500 volts, respectively. The nebulizer pressure was set at 40 psig.

## 3. Results

### 3.1. CRV431 Metabolic Stability in Human Liver Microsomes

**Figure 1:**
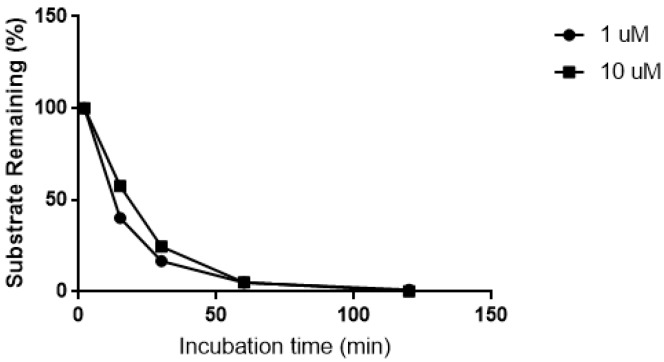
Biotransformation of CRV431 (1 and 10 μM) in human liver microsomes.

The biotransformation of CRV431 was nearly complete after 60 min incubation in human liver microsomes (Figure 1). In the absence of microsomal proteins and the NADPH-generating system, no biotransformation was observed over the same time period.

### 3.2. Cytochrome P450 Metabolism of CRV431 Using Recombinant Human CYP Enzymes

**Figure 2:**
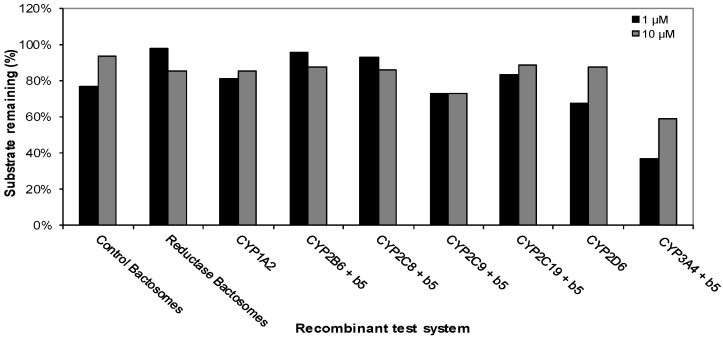
Metabolism of CRV431 (1 and 10 μM) by a panel of recombinant human CYP enzymes (50 pmol/mL).

This experiment was carried out to determine the cytochrome P450 (CYP) enzymes capable of metabolizing CRV431. The disappearance of CRV431 (1 μM) was observed in incubations with recombinant human CYP1A2 (19%), CYP2C9 (27%), CYP2C19 (17%), CYP2D6 (32%), and CYP3A4 (64%), while incubations with control bactosomes resulted in 24% loss. The loss of CRV431 (10 μM) was observed in incubations with recombinant human CYP2C9 (27%) and CYP3A4 (41%). Incubations with the other recombinant human CYP enzymes evaluated resulted in less than 15% substrate loss (Figure 2).

### 3.3. Cytochrome P450 Metabolism of CRV431 Using Chemical Inhibitors

**Figure 3:**
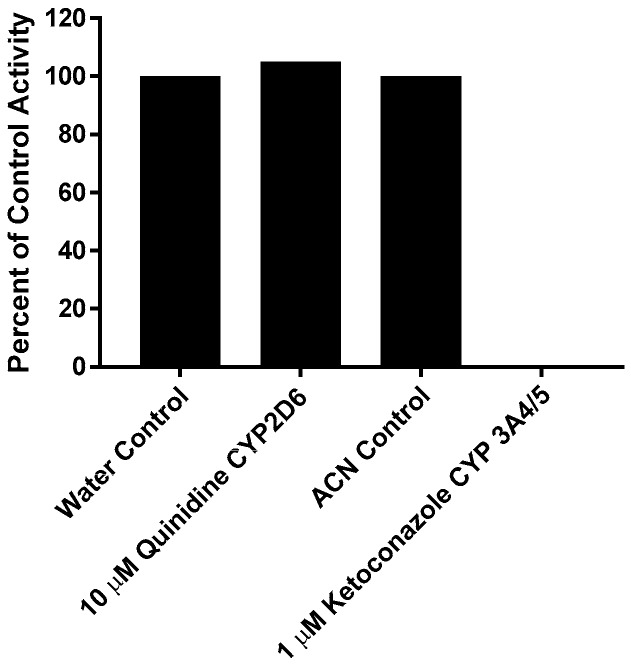
Effect of direct-acting chemical inhibitors on the loss of CRV431 (1 μM) by human liver microsomes.

This experiment was carried out manually to verify the role of individual CYP enzymes in the metabolism of CRV431. The effect of the direct-acting inhibitors on the loss of CRV431 by human liver microsomes is shown in Figure 3. Percent inhibition was calculated by the comparison of substrate loss in samples containing chemical inhibitors to respective solvent control samples incubated for the same interval. The inhibition of CRV431 loss by direct-acting inhibitors was observed in incubations with ketoconazole (CYP3A4/5 inhibitor, 100% inhibition). Incubations with quinidine (CYP2D6 inhibitor) showed no inhibition.

**Figure 4:**
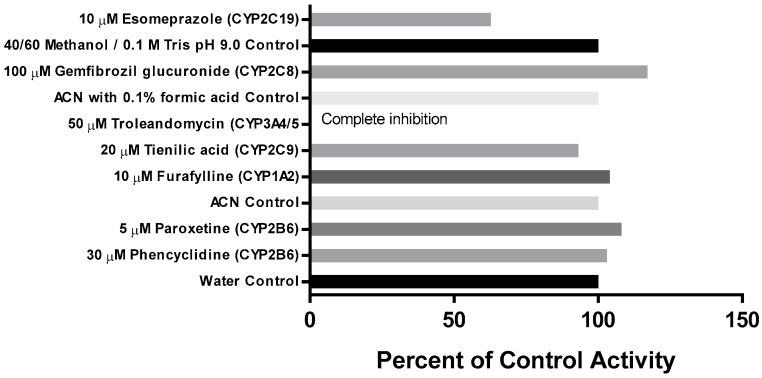
Effect of metabolism-dependent chemical inhibitors on the loss of CRV431 (1 μM) by human liver microsomes after 30-min preincubation.

The effect of the metabolism-dependent inhibitors on the loss of CRV431 by human liver microsomes is summarized in Figure 4. The inhibition of CRV431 loss by metabolism-dependent inhibitors was observed in incubations with tienilic acid (CYP2C9 inhibitor, 7% inhibition), esomeprazole (CYP2C19 inhibitor, 37% inhibition), and troleandomycin (CYP3A4/5 inhibitor, 100% inhibition). It is to be noted that esomeprazole is a mechanism-based inhibitor of both CYP2C19 and CYP3A4/5. Incubations with the other metabolism-dependent inhibitors evaluated resulted in no inhibition.

### 3.4. Liver Microsome Viability for Metabolic Profiling Studies

Incubations of verapamil with human, monkey, and rat liver microsomes demonstrated rapid metabolism. The verapamil half-life in all of the microsome incubations was less than 10 min. This is in line with the metabolism rate for verapamil [9], and confirms the viability of the human, monkey, and rat liver microsomes used in this study to assess the in vitro metabolism of CRV431.

### 3.5. CRV431 Metabolite Identification in Human Liver Microsomes

**Figure 5:**
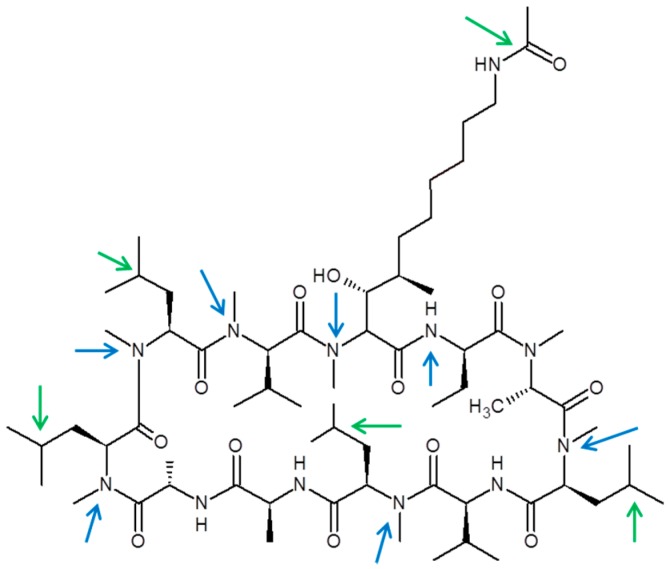
Chemical structure of CRV431 and proposed biotransformation sites. Potential oxidation (green arrows) and demethylation (blue arrows) sites are shown.

**Figure 6:**
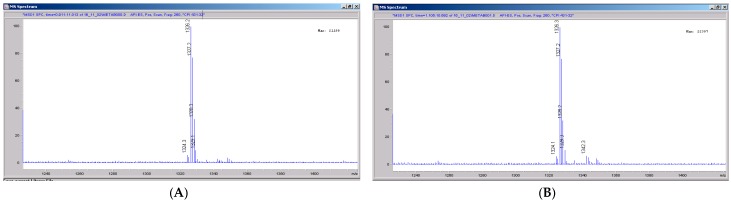
Mass spectral scans of human liver extracts incubated with CRV431. Human liver microsomes (1 mg/mL) were incubated with 1 μg/mL CRV431, and extracts were removed for analysis by electrospray ionization liquid chromatography mass spectrometry (ESI-LCMS) at 20 min (panel (**A**)), mass spectral scan from 1260–1460 *m*/*z* and 80 min (panel (**B**)), and mass spectral scan from 1260–1460 *m*/*z* (not shown).

**Figure 7:**
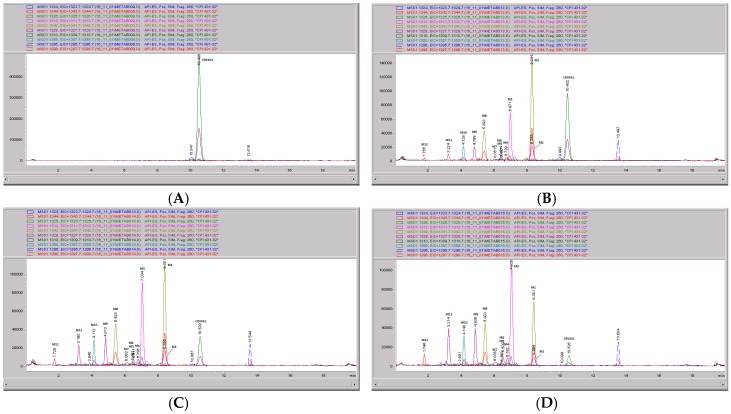
CRV431 metabolism in human liver microsomes. Human liver microsomes (1 mg/mL) were incubated with 1 ug/mL CRV431, and extracts were removed at 0 (panel **A**), 20 min (panel **B**), 40 min (panel **C**), and 80 min (panel **D**) for analysis by ESI-LCMS. Each liquid chromatography—mass spectrometry (LC–MS) profile displays the metabolite abundances as a function of chromatographic retention time.

The chemical structure of CRV431 and its potential biotransformation sites are indicated in Figure 5. The mass spectral scans for CRV431 incubated with human liver microsomes are shown in Figure 6, panels A and B. These low sensitivity scans allow for the identification of all metabolite masses. In addition to CRV431, 12 distinct masses were identified and subsequently used for more sensitive selected-ion monitoring of all CRV431 microsomal samples. The individual LC-MS metabolism profiles (0–80 min) for incubation with 1 μg/mL CRV431 are shown in Figure 7, panels A to D. The incubation of human liver microsomes with 1 μg/mL CRV431 for 0–80 min allowed for the identification of 12 distinct metabolites (M1–M12) resulting from various hydroxylation and demethylation reactions (Table 4), which are consistent with the metabolism previously reported with cyclosporine and analogues by the cytochrome P450 enzyme system [2,3,4]. The metabolite M4 is included for completeness, but likely arises from the demethylation of unsaturated CRV431, which is present as an impurity.

### 3.6. CRV431 Metabolite Identification in Monkey Liver Microsomes

**Figure 8:**
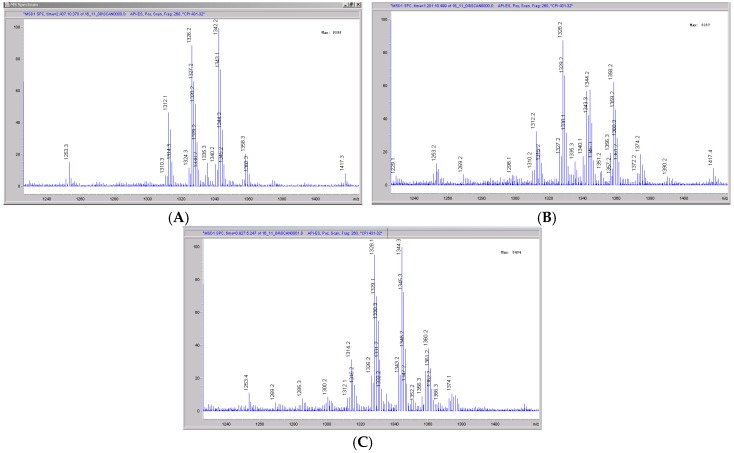
Mass spectral scans of monkey liver extracts incubated with CRV431. Monkey liver microsomes (1 mg/mL) were incubated with 1 μg/mL CRV431, and extracts were removed for analysis by ESI-LCMS at 5 min (panel (**A**), mass spectral scan from 1260–1460 *m/z*), 20 min (panel (**B**), mass spectral scan from 1260–1460 *m/z*) and 80 min (panel (**C**), mass spectral scan from 1260–1460 *m/z*).

**Figure 9:**
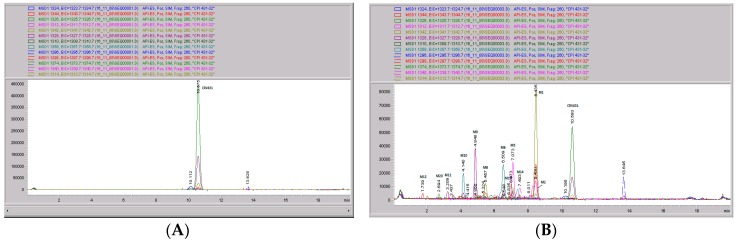

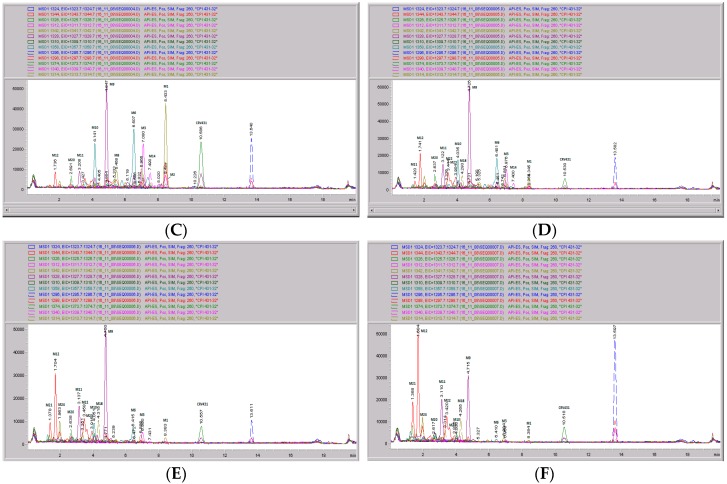
CRV431 metabolism in monkey liver microsomes. Monkey liver microsomes (1 mg/mL) were incubated with 1 ug/mL CRV431, and extracts were removed at 0 (panel (**A**), 5 min (panel (**B**)), 10 min (panel (**C**), 20 min (panel (**D**), 40 min (panel (**E**), and 80 min (panel (**F**) for analysis by ESI-LCMS. Each LC-MS profile displays the metabolite abundances as a function of chromatographic retention time.

The mass spectral scans for CRV431 incubated with monkey liver microsomes are shown in Figure 8, panels A to C. These low sensitivity scans allow for the identification of all metabolite masses. In addition to CRV431, three distinct masses were identified and subsequently used for more sensitive selected-ion monitoring of all CRV431 microsomal samples. The individual LC-MS metabolism profiles (0–80 min) for incubation with 1 μg/mL CRV431 are shown in Figure 9, panels A to F. The incubation of monkey liver microsomes with 1 ug/mL CRV431 for 0–80 min allowed for the identification of all 12 metabolites (M1–M12) detected in human liver microsomes. An additional 12 metabolites not observed in human liver microsomes were also seen (M13–M24) (Table 5). The rate of CRV431 metabolism in monkey liver microsomes was significantly greater (at least five-fold) than that seen in human microsomes. At 10 min incubation, the amount of CRV431 remaining in the human and monkey liver microsomes was 71.8% and 13.2%, respectively. With the exception of M15, all of the additional metabolites appeared to be a result of the further metabolism of M1–M12 (di- and tri-hydroxylations and demethylations), which was consistent with the higher metabolism rate.

### 3.7. CRV431 Metabolite Identification in Rat Liver Microsomes

**Figure 10:**
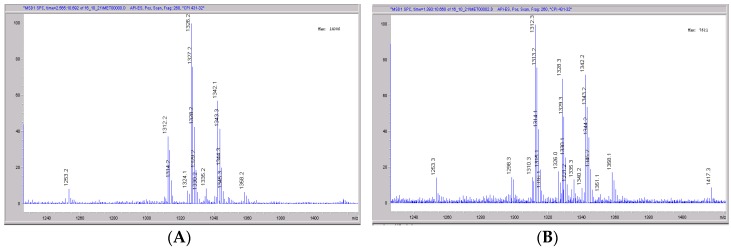
Mass spectral scans of rat liver extracts incubated with CRV431. Rat (Sprague-Dawley) liver microsomes (1 mg/mL) were incubated with 1 μg/mL CRV431, and extracts were removed for analysis by ESI-LCMS at 20 min (panel (**A**), mass spectral scan from 1260–1460 *m/z*) and 80 min (panel (**B**), mass spectral scan from 1260–1460 *m/z*).

**Figure 11:**
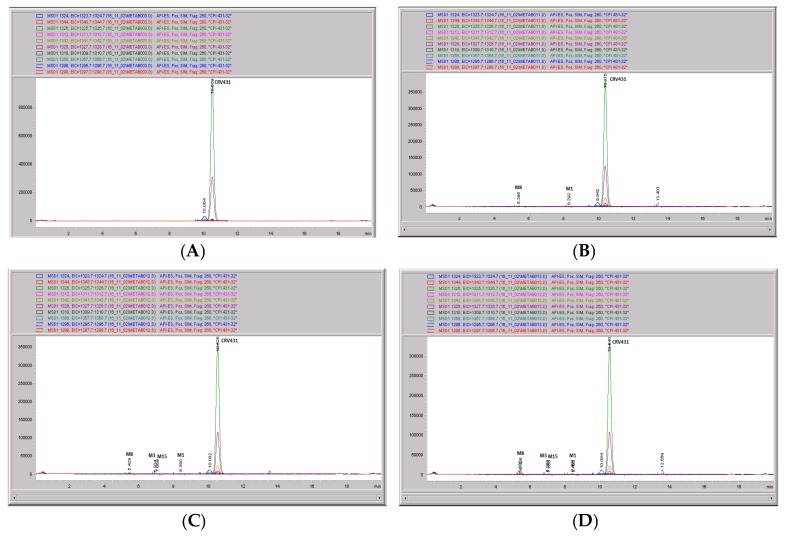
CRV431 metabolism in rat liver microsomes. Rat (Sprague Dawley) liver microsomes (1 mg/mL) were incubated with 1 ug/mL CRV431, and extracts were removed at 0 (panel (**A**)), 20 min (panel (**B**)), 40 min (panel (**C**)), and 80 min (panel (**D**)) for analysis by ESI-LCMS. Each LC-MS profile displays the metabolite abundances as a function of chromatographic retention time.

The mass spectral scans for CRV431 incubated with rat liver microsomes are shown in Figure 10, panels A and B. These low-sensitivity scans allow for the identification of all of the metabolite masses present. In addition to CRV431, three distinct masses were identified and subsequently used for more sensitive selected-ion monitoring of all of the CRV431 microsomal samples. The individual LC-MS metabolism profiles (0–40 min) for incubation with 1 μg/mL CRV431 are shown in Figure 11, panels A to D. The incubation of rat liver microsomes with 1 ug/mL CRV431 for 0–80 min allowed for the identification of only four metabolites: M1, M3, M8, and M15 (Table 6). The rate of CRV431 metabolism in rat liver microsomes was significantly less (15-fold) than that seen in human microsomes. At 80 min incubation, the amount of CRV431 remaining in the human and rat liver microsomes was 5.3% and 92.3%, respectively. This slow rate of metabolism has previously been observed for cyclosporine A and other cyclosporine-based analogues in rat microsomes [personal communication].

## 4. Conclusions and Discussion

The extensive hepatic metabolism of CsA results in three primary metabolites: the hydroxylated metabolites AMI and AM9, and the *N*-demethylated metabolite AM4n [6]. Cytochromes P450 3A4/5 are acknowledged to be predominantly responsible for the metabolism of CsA [7,8], although other cytochromes such as 3A9 may also play a role [10]. In this study, the in vitro metabolism of the CsA analogue CRV431 was shown to be NADPH-dependent in incubations with human liver microsomes. Incubations with recombinant human CYPs indicated that both CYP2D6 and CYP3A4 were able to significantly metabolize CRV431; however, in subsequent chemical inhibition (direct and metabolism-based) experiments with human liver microsomes, only CYP3A4 demonstrated significant activity. The cytochrome P450 experiments collectively indicate that CYP3A4 appears to be the predominant CYP enzyme that plays a role in the metabolism of CRV431 under the conditions evaluated.

The incubation of human liver microsomes with CRV431 allowed for the identification of 12 distinct metabolites (M1–M12) resulting from various hydroxylation and demethylation reactions (Figure 4), which is consistent with the metabolism reported for cyclosporine and analogues by the cytochrome P450 enzyme system. Monkey microsomes produced all 12 metabolites seen in human microsomes. Owing to the five-fold greater metabolism rate in monkey liver microsomes relative to human liver microsomes, an additional 12 metabolites (M13–M24) were also identified. The monkey is therefore a relevant animal species in which to study the preclinical metabolism of CRV431. Rat microsomes were very slow metabolizers of CRV431 (at least 15-fold less than human), and only M1, M3, M8, and M15 were detected.

CRV431 is being developed as a drug candidate for the treatment of certain viral infections, including hepatitis B. The current standard of care for the treatment of hepatitis B includes the use of nucleotide drugs such as tenofovir and entecavir [11]. It is anticipated that the drug–drug interaction potential between CRV431 and the nucleotide drugs used in the treatment of viral infections including hepatitis B would be minimal, as nucleotides are predominantly excreted renally, and not appreciably metabolized via cytochrome P450 enzymes.

## Figures and Tables

**Table 1:** LC-MS/MS Gradient Conditions for CRV431 Quantitation.

Time (min)	95:5 *v*/*v* Water: Methanol * (%)	Methanol *	Flow Rate (mL/min)
0.00	55	45	0.5
0.2	55	45	0.5
3.0	5	95	0.5
3.5	5	95	0.5
3.51	55	45	0.5
4.2	Stop	Stop	0.5

* also contains 1 mM ammonium acetate.

**Table 2:** LC-MS/MS Gradient Conditions for Elution of CRV431 and Metabolites.

Time (min)	dH20 * (%)	ACN *	Flow Rate (mL/min)
0.00	55	45	1.0
16.0	25	75	1.0
16.1	0	100	1.0
18.1	0	100	1.0
18.2	55	45	1.0

* also contains 0.02% Glacial acetic acid + 20 μM Sodium Acetate.

**Table 3:** LC-MS Gradient Conditions for Elution of Verapamil.

Time (min)	dH20 * (%)	ACN *	Flow Rate (mL/min)
0.00	70	30	1.0
8.0	45	55	1.0
8.1	0	100	1.0
10.1	0	100	1.0
10.2	70	30	1.0

* also contains 0.02% Glacial acetic acid + 20 μM Sodium Acetate.

**Table 4:** Identification of CRV431 metabolites in human liver microsomes. Human liver microsomes (1 mg/mL) were incubated with 1 ug/mL CRV431, and extracts were removed for analysis by ESI-LCMS.

Component	Proposed Biotransformation	Relative LC-MS Retention	*m*/*z*	Δ *m*/*z*	% of Total Drug-Related Mass Versus Time (minutes)				
					0 min	10 min	20 min	40 min	80 min
CRV431	NA	1.0	1326	0	96.4	57.0	27.5	10.4	4.0
CRV431 unsaturated impurity	NA	0.96	1324	NA	3.6	2.9	1.8	0.7	0.2
M1	Hydroxylation	0.8	1342	+16	0	21.2	28.3	27.4	18.3
M2	Demethylation	0.8	1312	−14	0	3.6	4.9	4.6	3.1
M3	Demethylation	0.67	1312	−14	0	5.3	13.8	21.9	27.4
M4	Demethylation of unsaturated CRV431	0.65	1310	−16	0	0.3	1.1	1.7	2.0
M5	Didemethylation	0.62	1298	−28	0	0	0.4	1.3	2.5
M6	Dihydroxylation	0.62	1358	+32	0	0	1.3	1.4	1.1
M7	Demethylation + Hydroxylation	0.58	1328	+2	0	0.1	0.6	1.2	1.7
M8	Hydroxylation	0.52	1342	+16	0	5.9	10.3	13.0	13.5
M9	Demethylation + Hydroxylation	0.47	1328	+2	0	1.1	3.2	5.6	8.6
M10	Dihydroxylation	0.39	1358	+32	0	1.2	3.3	5.3	6.6
M11	Demethylation + Hydroxylation	0.30	1328	+32	0	0.4	1.6	4.4	8.9
M12	Dihydroxylation + demethylation	0.17	1344	+18	0	0.1	0.5	1.0	1.9

**Table 5:** Identification of CRV431 metabolites in monkey liver microsomes. Monkey liver microsomes (1 mg/mL) were incubated with 1 ug/mL CRV431, and extracts were removed for analysis by ESI-LCMS.

Component	Proposed Biotransformation	Relative LC-MS Retention	*m/z*	Δ *m/z*	% of Total Drug-Related Mass Versus Time (minutes)						
					0 min	2.5 min	5 min	10 min	20 min	40 min	80 min
**CRV431**	NA	1.0	1326	0	96.3	59.4	21.5	12.3	4.3	6.6	6.7
**CRV431 unsaturated impurity**	NA	0.96	1324	−2	3.7	3.8	1.7	0.8	0	0	0
**M1**	Hydroxylation	0.8	1342	+16	0	18.1	24.2	16.1	3.2	1.7	0.9
**M2**	Demethylation	0.8	1312	−14	0	3.1	6.7	2.7	0.7	0	0
**M3**	Demethylation	0.67	1312	−14	0	3.8	7.6	8.3	5.4	3.3	0.7
**M4**	Demethylation of unsaturated CRV431	0.65	1310	−16	0	0.3	0.5	0.6	0.6	0	0
**M5**	Didemethylation	0.62	1298	−28	0	0	0.2	0.4	0.7	0.6	0
**M6**	Dihydroxylation	0.62	1358	+32	0	2.4	8.2	12.6	10.9	4.8	1.3
**M7**	Demethylation + Hydroxylation	0.58	1328	+2	0	0	0	1.1	0	0	0
**M8**	Hydroxylation	0.52	1342	+16	0	1.9	3.7	3.7	2.0	0	0.4
**M9**	Demethylation + Hydroxylation	0.47	1328	+2	0	1.9	8.6	15.6	24.8	25.2	17.6
**M10**	Dihydroxylation	0.39	1358	+32	0	0.8	4.4	6.9	6.7	4.2	1.4
**M11**	Demethylation + Hydroxylation	0.30	1328	+32	0	0.2	1.3	2.8	6.5	8.7	11.7
**M12**	Dihydroxylation + demethylation	0.17	1344	+ 18	0	0.1	0.7	1.8	6.2	11.7	21.6
**Additional Metabolites Not Detected in Human Liver Microsome Experiments**											
**M13**		0.75	1340	+14	0	1.1	1.6	1.1	0	0	0
**M14**		0.70	1340	+14	0	0.9	2.4	3.2	1.7	0.6	0
**M15**	Hydroxylation	0.65	1342	+16	0	1.6	3.3	3.9	3.3	2.4	1.7
**M16**	Trihydroxylation	0.50	1374	+48	0	0.4	1.8	2.6	2.2	1.4	0
**M17**	Trihydroxylation	0.46	1374	+48	0		0.4	0.8	0.9	1.1	0
**M18**	Didemethylation + hydroxylation	0.41	1314	−12	0	0	0.2	0.6	2.3	4.1	5.4
**M19**	Didemethylation + hydroxylation	0.31	1314	−12	0	0	0.2	0.4	1.3	1.8	2.8
**M20**	Trihydroxylation	0.25	1374	+48	0	0.1	0.8	1.6	3.3	2.9	1.8
**M21**	Dihydroxylation + demethylation	0.13	1344	+18	0	0	0	0	1.2	3.7	8.0
**M22**	Dihydroxylation + demethylation	0.32	1344	+18	0	0	0	0	5.6	8.4	11.1
**M23**	Dihydroxylation + demethylation	0.35	1344	+18	0	0	0	0	3.6	2.6	1.4
**M24**	Hydroxylation	0.18	1342	+16	0	0	0	0	2.3	4.0	5.7

**Table 6:** Identification of CRV431 metabolites in rat liver microsomes. Rat liver microsomes (1 mg/mL) were incubated with 1 ug/mL CRV431, and extracts were removed for analysis by ESI-LCMS.

Component	Proposed Biotransformation	Relative LC-MS Retention	*m/z*	Δ *m/z*	% of Total Drug-Related Mass Versus Time (minutes)				
					0 min	10 min	20 min	40 min	80 min
CRV431	NA	1.0	1326	0	96.2	95.4	94.6	93.6	92.3
CRV431 unsaturated impurity	NA	0.96	1324	NA	3.8	3.9	3.8	3.9	4.6
M1	Hydroxylation	0.8	1342	+16	0	0.1	0.3	0.5	0.5
M3	Demethylation	0.67	1312	−14	0	0	0.07	0.19	0.4
M8	Hydroxylation	0.52	1342	+16	0	0.4	0.8	1.3	1.7
M15	Hydroxylation	0.65	1342	+16	0	0.08	0.1	0.2	0.2
